# Invisible battles: the disability paradox and workplace experiences of individuals with lupus

**DOI:** 10.3389/fpubh.2026.1781849

**Published:** 2026-03-31

**Authors:** Armand Bam, Liam Kilbride, Linda Ronnie

**Affiliations:** 1 Stellenbosch Business School, Stellenbosch University, Stellenbosch, Cape Town, South Africa; 2 Department of Psychology, Stellenbosch University, Stellenbosch, Cape Town, South Africa; 3 School of Management Studies, Faculty of Commerce, University of Cape Town, Rondebosch, Cape Town, South Africa

**Keywords:** inclusivity, invisible disability, paradox theory, social model of disability, Systemic Lupus Erythematosus

## Abstract

**Background:**

Individuals living with Systemic Lupus Erythematosus (SLE), experience complex interplay between fatigue, mental health challenges, and workplace inclusion. SLE-related fatigue extends beyond physical exhaustion, encompassing cognitive and emotional depletion, while anxiety and depression further compound its effects. By integrating Paradox Theory and the Social Model of Disability, this research conceptualizes how competing workplace demands and structural barriers contribute to employment instability and reinforce exclusion.

**Methods:**

A qualitative research design is employed, drawing from in-depth narrative interviews with six individuals living with SLE in South Africa.

**Results:**

Two critical themes emerged from the study: pervasive fatigue and mental health cycles. The findings underscore the need for structural changes that shift responsibility from individuals managing their symptoms to workplaces creating adaptable environments.

**Conclusion:**

Addressing these issues is essential for reducing stigma, promoting inclusivity, and ensuring equitable employment opportunities for individuals with invisible disabilities.

## 1 Introduction

Systemic Lupus Erythematosus (SLE) is a complex, chronic autoimmune disorder that affects millions of individuals worldwide. It is characterized by a diverse array of symptoms, including joint pain, skin rashes, organ damage and neurological complications (1, 2). While much of the literature has focused on the physiological manifestations of the disease (3, 4), the invisible burden it imposes on individuals' daily functioning, particularly in professional settings, remains significantly understudied. Fatigue is one of the most pervasive yet least understood symptoms of SLE, profoundly affecting cognition, emotional wellbeing and workplace participation (5, 6). Coupled with mental health challenges such as depression and anxiety, fatigue can create a cyclical pattern of depletion, further exacerbating employment instability and workplace exclusion.

Despite growing awareness of chronic illnesses in workplace discourse, individuals with SLE continue to encounter significant barriers to employment retention. Unlike visible disabilities, which often prompt immediate accommodations, the fluctuating and largely invisible nature of SLE symptoms makes it difficult for employers and colleagues to recognize the extent of their impact (7, 8). As a result, individuals with SLE frequently navigate a paradox: whether to disclose their condition in the hope of securing accommodations or to conceal it to avoid stigma, discrimination and career setbacks (9). This paradox of disclosure vs. concealment is further compounded by the pressure to maintain productivity in environments that privilege consistency and reliability, reinforcing an ableist model of work (10, 11).

Paradox Theory (12) provides a lens through which to understand how workplace structures and cultural norms shape the experiences of individuals with SLE by identifying the competing demands placed on individuals balancing productivity with health management, independence with the need for support, and resilience with vulnerability. These paradoxes are not simply personal dilemmas but are produced and reinforced by structural inequities in the workplace. The Social Model of Disability (13, 53) allows for further contextualization of these challenges by shifting the focus from individual impairment to the systemic barriers that exclude individuals with chronic illnesses. Rather than positioning disability as a medical deficit to be managed, this framework highlights how rigid workplace policies, skepticism toward invisible conditions, and the absence of inclusive leadership contribute to employment precarity for individuals with SLE.

By exploring the lived experiences of individuals with SLE, this study aims to deepen employers' understanding of the interplay between chronic illness, fatigue, mental health and workplace inclusion. The study examines the complex ways in which fatigue and mental health cycles shape employment experiences, the dilemmas individuals face regarding disclosure and accommodation, and how workplace structures, organizational policies, and managerial practices enable or constrain the employment participation of individuals with SLE.

This research has important practical and policy implications, particularly for human resource management, workplace inclusion strategies and leadership development. Addressing the employment challenges faced by individuals with SLE requires a reimagining of workplace norms to accommodate the fluctuating nature of chronic illness, promote psychological safety, and challenge ableist productivity expectations.

## 2 Theoretical background

### 2.1 Chronic illness and invisible disabilities in the workplace

Invisible disabilities, which refer to physical, cognitive or psychological conditions that are not immediately apparent but significantly impact daily functioning (14), present unique challenges in professional settings. Unlike visible disabilities, which often prompt immediate accommodations, invisible conditions frequently remain unrecognized, leading to stigma, misunderstanding and inadequate workplace adjustments (7, 8). Employees with these conditions must navigate the difficult decision of whether to disclose their health status, weighing the potential benefits of support against the risks of discrimination (9).

Invisible disabilities include chronic illnesses such as SLE, multiple sclerosis, fibromyalgia and chronic fatigue syndrome, as well as autoimmune diseases like rheumatoid arthritis, neurological disorders like epilepsy and migraines, and mental health conditions such as depression and anxiety disorders (15). Employees with such conditions often encounter barriers to inclusion because their symptoms are not visibly evident, making it challenging for employers and colleagues to understand their limitations (10). SLE exemplifies the complexity of managing an invisible illness in the workplace. Individuals with SLE frequently experience unpredictable symptoms such as fatigue, joint pain, cognitive dysfunction (commonly referred to as “lupus fog”) and heightened susceptibility to infections (16). Workplace challenges for individuals with invisible disabilities are compounded by misconceptions and skepticism about their conditions. Because their symptoms fluctuate and are not outwardly visible, employers may perceive affected employees as unreliable which further complicates job retention (17).

Employees with chronic illnesses often encounter doubt regarding the legitimacy of their symptoms. Supervisors and colleagues may assume that because an individual appears healthy, they are not struggling (8). This skepticism leads to what scholars describe as “benevolent marginalization,” wherein employers express sympathy but fail to take meaningful action to accommodate workers' needs (11). Unlike visible disabilities that require concrete accommodations such as wheelchair accessibility, individuals with invisible illnesses often need flexible schedules, workload adjustments, remote work options or frequent rest breaks, accommodations that many employers are reluctant to implement (9). Studies indicate that employees who successfully maintain their jobs despite chronic illness often rely on informal support networks rather than official workplace accommodations, highlighting the insufficiency of current disability policies (15).

Disclosure of invisible disabilities remains a complex and distressing decision. Many employees fear that revealing their condition will result in job loss, demotion or social exclusion (7). Conversely, those who disclose their condition to supportive employers often report higher job satisfaction and improved performance (18). However, the fear of being stigmatized prevents many from seeking the accommodations they need, resulting in further workplace struggles.

Invisible disabilities have a profound impact on employment outcomes due to stigma, fluctuating symptoms and inadequate formal accommodations (19). Addressing these challenges requires proactive employer engagement, adaptable workplace policies and a commitment to fostering inclusivity (55). Organizations that prioritize employee wellbeing and workplace flexibility will not only enhance productivity but also contribute to a more equitable and supportive professional environment for all workers, regardless of whether their disabilities are visible or invisible (7, 10).

### 2.2 Fatigue and health

Fatigue and mental health present additional, unique barriers in the workplace (54). Chronic fatigue, one of the most disabling yet least understood symptoms of conditions like SLE, is not relieved by rest; affects productivity, cognitive function, and concentration; and can ultimately force individuals out of employment (20). The mental health burden of managing an invisible disability at work includes anxiety, stress and depression, particularly in environments where employees feel pressured to “prove” their condition (7). Employees with chronic illnesses frequently experience heightened workplace anxiety, especially concerning job security. Many take pay cuts, avoid promotions or switch to part-time roles to manage their health effectively (10).

Addressing these challenges requires targeted workplace strategies. Employers must educate themselves and their staff on invisible disabilities to foster a culture of understanding and empathy (7). Workplace disability awareness programs can play a significant role in reducing stigma and correcting misconceptions about conditions like SLE Fatigue and health. Implementing flexible work arrangements, such as remote work options, adaptable schedules and job-sharing, can significantly improve working conditions for employees with fluctuating symptoms (21). Providing employees with customized accommodations, including frequent breaks or quiet workspaces, enables them to manage their symptoms effectively without compromising productivity (22, 23).

Formalizing workplace support systems is another crucial step toward fostering an inclusive work environment. Peer support groups and mentorship programs for employees with chronic illnesses can provide essential guidance and solidarity (24). Organizations must also ensure that workplace accommodations are applied consistently and equitably, rather than left to the discretion of individual managers (25).

### 2.3 Fatigue as a multifaceted challenge

Fatigue is a pervasive and multidimensional symptom affecting individuals across various conditions. However, for those with chronic illnesses like SLE, fatigue presents as a persistent, unpredictable and often debilitating experience that significantly impacts daily functioning, employment and overall quality of life. Unlike general tiredness, which resolves with rest, SLE-related fatigue is an overwhelming state of exhaustion that does not follow a natural circadian rhythm and is resistant to conventional recovery mechanisms (26).

Fatigue is often conceptualized as an interplay of physical, mental, and emotional exhaustion (27). Physiologically, it is linked to markers such as decreased neural efficiency, glucose depletion and metabolic dysregulation. Psychologically, fatigue is associated with stress, anxiety and depression, which are often exacerbated by chronic conditions (28). Emotionally, it leads to burnout, withdrawal and a reduced sense of wellbeing (29).

A critical challenge in defining fatigue is its variability across individuals and contexts. Skau et al. (30) argue that narrow definitions of fatigue, whether physical, cognitive or emotional, fail to capture its complexity. Instead, fatigue should be understood as a state resulting from different forms of exertion (e.g., sleep-related exertion, task-related exertion and cognitive overload). Conservation of Resources (COR) theory posits that emotional exhaustion, a key component of fatigue, emerges from the loss or perceived loss of vital psychological and social resources. This depletion leads to diminished job performance and eventual withdrawal from work (31).

A fundamental distinction between general tiredness and chronic illness-related fatigue is the nature of its onset, persistence and resistance to recovery. General fatigue, which is experienced by healthy individuals and follows a circadian rhythm energy depletion, is followed by recuperation through sleep and rest (32). In contrast, chronic fatigue in SLE patients operates independently of natural recovery cycles, often leaving individuals in a state of unrelenting exhaustion.

Studies on cancer-related fatigue provide insights into chronic fatigue syndromes. Glaus et al. (33) compared fatigue in healthy individuals vs. cancer patients, showing that while healthy individuals experience fatigue as a protective mechanism that promotes recovery, cancer patients describe fatigue as an ever-present state that is unresponsive to rest. The same principle applies to SLE where the body's ongoing inflammatory response depletes energy reserves even in the absence of physical exertion (27).

In SLE, fatigue is further compounded by co-morbid conditions such as hypothyroidism, anemia and fibromyalgia, each of which can contribute to exhaustion independently. Cleanthous et al. (27) conducted a meta-analysis of 55 studies, concluding that 67%−90% of SLE patients experience debilitating fatigue. The bi-directional nature of SLE-related fatigue means that as fatigue worsens, mental health conditions such as anxiety and depression increase, further exacerbating exhaustion.

Neuropsychiatric research supports this link between fatigue and cognitive dysfunction: SLE patients with higher inflammatory markers, such as IL-6 and B-lymphocyte stimulators, exhibit greater cognitive impairment and sustained attention deficits (34). Brain imaging studies indicate structural and functional changes in the prefrontal cortex and caudate nucleus, regions responsible for executive functioning, attention and mood regulation, suggesting that fatigue in SLE is not merely a symptom but a neurologically embedded condition.

Fatigue in SLE has profound implications for employment and overall wellbeing. Booth et al. (10) conducted a large-scale survey and found that 40%−45% of individuals with SLE were unable to sustain employment due to fatigue and symptom fluctuations?. The primary barriers to employment included:

- unpredictability of fatigue, leading to inconsistent work performance;
- cognitive dysfunction, making concentration and decision-making difficult;
- fear and anxiety regarding job security and financial stability; and
- lack of workplace accommodations as many employers do not recognize invisible disabilities.

The emotional burden of job insecurity, compounded by financial stress, results in increased anxiety and depressive symptoms among SLE sufferers (35). Studies indicate that depression and anxiety are twice as prevalent among SLE patients compared to the general population, further reducing motivation and workplace participation. The relationship between workplace accommodations and job retention is well-documented. McDowell and Fossey (36) emphasized that workplace adjustments such as flexible scheduling, modified training and role adaptations can significantly improve job retention among individuals with chronic illnesses. However, many workplaces lack awareness or willingness to implement these adjustments.

Beyond employment, fatigue significantly affects social participation and quality of life. Calderón et al. (37) explored the interplay between cognitive impairment, depression and disease activity in SLE patients, finding that executive dysfunction and depressive symptoms were the strongest predictors of diminished quality of life. Their study underscored the need for holistic interventions that address both physical and mental aspects of fatigue. Fatigue in SLE is not merely a byproduct of the disease but a central, debilitating feature that influences nearly every aspect of daily life. Unlike general tiredness, SLE-related fatigue is persistent, resistant to rest, and compounded by co-morbid conditions. The cognitive and emotional toll of fatigue disrupts employment, social participation and psychological wellbeing. Current workplace policies often fail to accommodate the needs of individuals with chronic illnesses exacerbating financial and emotional stress.

### 2.4 Paradox theory and the social model of disability

The workplace experiences of individuals with SLE are shaped by a complex interplay of competing demands and structural barriers. While the physical and mental health challenges of SLE significantly impact professional life, the broader issue is not merely the illness itself but the way in which workplaces respond to it. Paradox Theory and the Social Model of Disability together provide a comprehensive framework for understanding these challenges, illustrating the tensions faced by individuals with invisible disabilities and the systemic barriers that either hinder or facilitate their inclusion.

Paradox Theory provides a valuable lens for understanding the complex tensions faced by individuals with SLE in the workplace. According to Smith and Lewis (12), paradoxes represent persistent contradictions between interdependent elements. In the context of SLE, this manifests in several key ways. Employees with SLE often face competing demands between managing their health needs and meeting workplace expectations, creating what Waldman et al. (38) describe as tension between opposing yet interrelated forces. The Social Model of Disability, as articulated by Oliver (13, 39), shifts focus from individual impairment to how societal structures and attitudes create barriers to participation. This framework is particularly relevant for SLE, as an invisible disability marked by fluctuating symptoms (10).

The intersection of these theoretical frameworks reveals several critical insights. First, they highlight what could be termed the “disclosure paradox”—individuals with SLE must navigate whether to disclose their condition to receive accommodations while risking stigma or discrimination. This reflects the broader tension identified by Booth et al. (10) between visibility and invisibility in managing chronic illness at work. Second, these frameworks expose the “accommodation paradox” where workplace adjustments, while necessary, can inadvertently reinforce perceptions of difference or inability. This connects to what Goering (39) describes as the need to reconceptualize disability as a product of social arrangements rather than individual limitations. For SLE individuals, whose symptoms like fatigue and cognitive impairment can significantly impact work performance (37), this tension is particularly acute.

The “flexibility paradox” represents another key tension, where the need for adaptable working arrangements conflicts with organizational demands for consistency and predictability. Cleanthous et al. (27) research on fatigue in SLE patients demonstrates how symptoms can fluctuate unpredictably, making standard work schedules particularly challenging. The integration of these theoretical perspectives also illuminates what Ubel et al. (40) term the “disability paradox”—the disconnect between how others perceive the impact of chronic illness and how individuals with the condition experience it. For SLE employees, whose quality of life is affected by multiple, often invisible symptoms (6), this paradox can manifest in workplace interactions where the severity of their condition is underestimated or misunderstood.

Understanding these paradoxes through the lens of the social model reveals how workplace challenges for individuals with SLE stem from systemic barriers that Oliver (13) identifies as the root cause of workplace exclusion, rather than individual limitations. The fatigue and mental health cycles documented by Calderón et al. (37) and Caruso Mazzolani et al. (6) interact with workplace structures in ways that can either exacerbate or alleviate exclusion. This aligns with Smith and Lewis (12) assertion that paradoxes require organization-level responses rather than individual coping mechanisms. The integration of Paradox Theory with the Social Model of Disability helps explain why traditional approaches to workplace accommodation often fall short as they focus on individual adaptations and in effect, create unnecessary barriers to full participation.

This theoretical framework suggests that meaningful change requires a fundamental shift in how organizations approach chronic illness in the workplace. Rather than viewing the disclosure paradox, productivity tensions and flexibility challenges as individual problems to be managed, they must be recognized as structural issues requiring systemic solutions. As Goering (39) argues, this involves reimagining workplace norms to accommodate the realities of chronic illness rather than expecting individuals to conform to rigid structures. In this way, the dilemmas surrounding disclosure and accommodation become opportunities for organizational learning and transformation, rather than individual struggles.

By examining these issues through the dual lenses of Paradox Theory and the Social Model of Disability, this study aims to obtain a better understanding of how fatigue and mental health cycles interact with workplace structures to shape employment experiences. The study therefore explores the lived experiences of individuals with SLE while also providing a theoretical foundation for developing more inclusive workplace practices. The objective is not merely to help individuals navigate existing barriers but to transform workplaces into spaces where chronic illness is understood and accommodated as part of human diversity, enabling full participation and contribution from all employees.

## 3 Methods

To investigate the experiences of individuals living with SLE in South Africa, this study employed an interpretative multiple case study methodology. This approach was ideal for exploring complex phenomena within a real-world context (41). The methodology enabled a nuanced understanding of how participants navigated their professional responsibilities alongside personal health challenges. Each case was contextualized within the participant's unique workplace environment, emphasizing organizational structures, health policies and socio-environmental factors. South Africa's distinctive socio-legal landscape further highlighted the intersections between healthcare accessibility, labor policies and societal perceptions of chronic illness (42).

The following research questions guided the investigation:

1. How does fatigue, as experienced by individuals with SLE, affect their ability to engage in daily activities and maintain employment?
2. In which ways do mental health challenges, such as depression and anxiety, intersect with physical symptoms to create additional burdens for individuals living with SLE?
3. How do workplace structures, organizational policies, and managerial practices enable or constrain the employment participation of individuals with SLE?

### 3.1 Sample selection

Participants were identified through purposive sampling, targeting adults aged 18–65 living with SLE who were employed, transitioning jobs or seeking employment. A pilot case drawn from the researchers' networks served to refine the interview guide as recommended by Maxwell (43). Feedback from this pilot informed the final recruitment strategy, which leveraged partnerships with SLE-focused organizations to disseminate an invitation to participate in the study. Snowball sampling complemented this effort facilitating access to additional participants through referrals.

Six participants were ultimately selected to ensure rich, in-depth data collection, adhering to best practices in multiple case study research (44). This sample size aligns with the principle of theoretical saturation ensuring sufficient depth while maintaining analytic rigor (45).

### 3.2 Data collection methods

Data were collected through semi-structured interviews. As participants were offered flexibility, including multiple shorter sessions if needed (46), the interviews were conducted in two sessions per participant, each lasting approximately 90 min. Sessions were spaced approximately 1 week apart but adjusted for health or work constraints with a maximum interval of 3 weeks. The interview guide drew on Spradley (47) ethnographic framework, encompassing descriptive, structural and contrast questions. Topics covered the impact of SLE on daily activities, workplace dynamics and disclosure challenges. A specific focus was placed on understanding the interplay between fatigue, mental health and physical symptoms in shaping participants' lived experiences. Complementary data sources included organizational inclusion policies, statutory benefits and employer websites, enriching the contextual understanding of participant narratives. Observational notes captured non-verbal cues and situational nuances, adding depth to the dataset (48).

### 3.3 Analytical approach

Thematic analysis followed Braun and Clarke (49) six-phase framework. Familiarization involved repeated reading of transcripts and supplementary documents. Coding was conducted systematically, identifying key patterns and features across the data corpus. Themes were iteratively refined through collaborative review to ensure coherence and validity (50). Cross-case synthesis facilitated a comparative analysis, uncovering shared and divergent experiences among participants (51).

Atlas.ti (Lumivero, LLC, Germany) software streamlined data organization, enabling efficient theme development and cross-case comparisons. This process illuminated individual narratives while identifying broader patterns, particularly in relation to how fatigue and mental health challenges impacted participants' daily functioning and work life.

### 3.4 Ethical safeguards

Ethical approval was secured from the university's Research Ethics Committee. Informed consent was obtained from all participants prior to data collection and access to counseling services was offered to those needing support. The study design prioritized respect, sensitivity and participant wellbeing consistent with the ethical principles outlined by Beauchamp and Childress (56).

## 4 Results

The findings of this study are organized through two overarching themes from the thematic analysis: *pervasive fatigue* and *mental health cycles*. While these themes are presented as distinct, they are intertwined in the experiences of participants in a symbiotic manner. Critically, both themes are also shaped by the workplace structures, organizational cultures, and the systemic conditions participants navigated, and these structural dimensions are foregrounded throughout the analysis that follows. Brief vignettes of each participant are presented in Table 1 to provide an understanding of each participant's context and history.

**Table 1:** Participant descriptions.

Participant	Description
*James: A diagnosis shaped by financial instability psychological strain*	James, 41, reflected on the emotional toll of receiving a SLE diagnosis during a period of financial uncertainty. Diagnosed at 28 while temporarily unemployed, without health insurance, he initially sought care at a public hospital but was overwhelmed by the long wait times, distressing conditions. Witnessing a patient's death in the hospital heightened his anxiety, reinforcing his reluctance to stay. The experience left James feeling helpless, emotionally drained, leading him to delay seeking medical care. When he was finally diagnosed in a private healthcare facility, the relief was mixed with frustration over how financial insecurity had delayed his access to proper treatment. Since then, James has remained within the private healthcare system, but fatigue, the stress of maintaining financial stability to afford care continued to weigh on his mental wellbeing.
*Trish: A journey through public healthcare, the mental toll of delayed care*	Trish, 57, was diagnosed with SLE unexpectedly at the age of 37 following an unrelated surgical procedure. Without private health insurance, she relied on public healthcare, enrolled in a Lupus Clinic, where she remained for 14 years. While the structured program provided consistency, long waiting periods for specialist appointments, uncertainty regarding medication availability, the sheer exhaustion of navigating the system often left her feeling powerless. Managing fatigue was particularly challenging as public healthcare required frequent hospital visits, long commutes, emotional resilience to withstand the unpredictability of care. For years, the anxiety of not knowing whether she could access timely treatment weighed heavily on Trish's mental health. The transition to private healthcare later in life alleviated some of these concerns, but the years spent in an overstretched system continue to shape her outlook on managing lupus.
*Karen: Stability in healthcare access but an unseen mental burden*	Karen, 53, has had continuous access to private healthcare since her diagnosis at 28. With comprehensive medical insurance, she benefitted from uninterrupted specialist care, early medical interventions which allowed her to manage her SLE symptoms proactively. However, although she has not experienced systemic barriers, the long-term reality of living with a chronic illness had taken an emotional toll. The expectation that access to care should equate to better health outcomes have, at times, left her feeling frustrated when persistent fatigue, flare-ups disrupt her daily life. The psychological burden of maintaining a demanding career while managing an invisible illness has also contributed to cycles of stress, burnout. Despite the advantages of private healthcare, Julie acknowledges that medical access alone does not mitigate the mental health challenges that come with living with a chronic condition.
*Debbie: The role of privilege in managing lupus, mental exhaustion*	Debbie, 35, recognized that her privileged access to private healthcare had provided her with a level of security that many others with SLE do not have. Diagnosed at 21, she consistently had access to top-tier medical professionals, early interventions, a clear treatment plan. However, despite this stability, the nature of lupus itself, through unpredictable flares, chronic fatigue, invisible symptoms, made mental wellbeing a complex challenge. Debbie had often felt a disconnect between how others perceive her as ‘privileged', the reality of managing an illness that disrupts her life in ways that are not always understood. Fatigue remained a constant presence, affecting her productivity, social life, while the mental strain of navigating an illness that does not always have clear solutions had led to moments of deep frustration, exhaustion.
*Erica: A late diagnosis, the emotional burden of a chronic condition*	Erica, 43, was diagnosed with SLE at 36 after years of unexplained symptoms. Having always relied on private healthcare, she was able to access specialist care quickly after her diagnosis, but the years leading up to it were marked by confusion, self-doubt, medical gaslighting. The emotional impact of being dismissed by doctors before her formal diagnosis left her struggling with anxiety, self-advocacy fatigue. Since receiving a diagnosis, her access to private healthcare allowed her to manage the disease effectively, but the mental exhaustion of chronic illness persisted. The unpredictability of lupus symptoms affected her confidence in making long-term plans, reinforcing a lingering sense of uncertainty that impacted both her work, personal life.
*Kim: The struggles of public healthcare, the emotional cost of limited choices*	Kim, 38, had spent her entire lupus journey within the public healthcare system. Diagnosed at 23, her financial situation had not allowed her the option of private medical care. The exhaustion of navigating the public healthcare system – waiting months for appointments, dealing with medication shortages, having to advocate for herself in an underfunded system – had been compounded by the physical fatigue of LSE itself. Each hospital visit required extensive time, patience, energy that she often did not have. The stress of financial insecurity further exacerbated her condition, as she constantly worried about whether she would receive the care she needed. Mental health support was not always readily available in public healthcare, leaving her to manage both the psychological, physical burdens of lupus largely on her own.

Table 2 presents a structured summary of the findings emphasizing the interplay between pervasive fatigue and mental health cycles and their profound effects on the lives of individuals with SLE.

**Table 2:** Thematic table of findings: living with Systemic Lupus Erythematosus (SLE).

Theme	Sub-themes	Illustrative quotes	Impact/Implications	Paradox
Pervasive fatigue	Nature and perception of fatigue	Fatigue described as “absolute exhaustion” and “never goes away even if you have 20 hours of sleep” (Kim).	Severe impact on daily work and quality of life Misunderstanding in the workplace Struggle to communicate SLE's debilitating nature to others	Productivity-health paradox
	Physical manifestations	Tasks like “putting up my arms” were difficult (Erica). Medication offered temporary relief but introduced side effects like sleeplessness (Kim) and blackouts (Karen).	Reduction in independence Increased need for workplace accommodations such as reduced hours and remote work	Productivity-health paradox
	Workplace implications	Participants cloaked their fatigue to avoid stigma: “getting older” (Trish) or “working too hard” (James).	Perceived as inadequacy in workplace settings Employers often fail to understand the condition's effects	Disclosure-concealment paradox
	Emotional and psychological effects	Constant fatigue led to feelings of inadequacy, frustration and demoralization: “It doesn't matter how much I wanted to do it, I just couldn't” (Debbie) and “I wanted to go to work. I needed to go to work” (James).	Need to reevaluate priorities and accept limitations	Productivity-health paradox
	Coping mechanisms	Flexible work schedules and empathy from managers alleviated some burdens: “work half day” (Erica) and “on a more flexible schedule” (Debbie).	Highlighted the importance of supportive workplace environments for maintaining productivity and wellbeing.	Disclosure-concealment paradox
Mental health cycles	Depression and anxiety	Depression linked to SLE was prevalent: “In your heart, there are fears you normally don't describe to anybody” (Erica). Participants described isolation and a duality in existence (public vs. private self): “Lupus is a very lonely disease” (Karen) and “the face I show and the face I hide” (James).	Psychological struggles compounded physical symptoms Emotional burdens extended to families and colleagues	Disclosure-concealment paradox
	Mental health support	Collaboration with psychologists and support systems was crucial for managing emotional wellbeing: “I've spoken to doctors about medication, anti-depressants, and they are working in collaboration” (Debbie).	Need for holistic care approaches addressing both mental and physical health	Independence- support paradox
	Emotional burden of medication	Medication side effects exacerbated mental health challenges: “Prednisone was like 12-16 tablets a day” (Trish). The side effects are often not understood: “broken sleep was hectic in a high-performance environment” (James).	Medication management requires careful monitoring and workplace understanding of its effects	Productivity-health paradox
	Coping strategies	Participants sought purpose beyond illness: “You can't let your illness define you” (Debbie).	Building resilience through meaningful engagement in work and life activities helped participants maintain a sense of normalcy and purpose	Disclosure-concealment paradox
	Cyclical nature of mental and physical health struggles	Mental and physical effects are interconnected: “My lupus makes my depression worse, and my depression makes my lupus worse” (Kim).	Need for integrated care addressing the interplay between physical symptoms and mental health challenges	Independence- support paradox

### 4.1 Pervasive fatigue

Lupus fatigue is debilitating and pervasive and unlike general tiredness, it is extreme and without relief. The relentless nature of fatigue led to “*an absolute exhaustion”* that “*never goes away even if you have 20 hours of sleep”* according to Kim. Erica shared her similar experience and added that “*mentally you need to be very strong to go through this.”* Compared to other participants, James hid the source of his fatigue when he explained to co-workers that it had to do with “*getting older”* or that he was “*working too hard”* which took him to the point of burnout because his “*body can't take the hits like it used to.”*

This fatigue made daily work and social activities difficult and “*not even exercise can change it”* according to Erica. For all participants, there was a recognition, and sense of resignation, that lupus was something that could not be “walked off” or that sleep could not address it. It was a disease that affected work schedules and in severe case resulted in job losses. For Kim, one of the challenges was conveying the overwhelming nature of fatigue in the workplace which was not easy because “*people just don't understand.”* This communicative burden was further compounded by workplace norms that prized consistent output and penalized visible inconsistency, leaving participants without an organizational language or framework through which to legitimize their experience. Kim explained how people did not see the impact of her fatigue:

“*I do a 12- or 16-hour day, then go to bed and can't sleep. [When I] wake up in the morning I am still exhausted and have to do what I planned, and by the end, I am just more exhausted. It's a tiredness that's part of life.”*

The fatigue manifested physically, and the effort required to perform basic activities was perceived as monumental and at times futile. Erica explained that something as simple as “*putting up my arms”* became challenging “*because you are so tired.”* This experience led to one of the more common accommodation needs for employers to reduce work hours. For Karen, the fatigue directly impacted her mobility and while “*working fulltime, I got to the point where I could no longer drive myself to work.”* James shared that lower energy levels contributed to feelings of inadequacy, and he had to re-evaluate his priorities, making lifestyle adjustments and forgoing opportunities because it took “*longer to recover.”* For all participants the physical impact of fatigue reduced their independence.

“*Yesterday was an outlier day. I felt so ill, I phoned my parents and said, ‘I need to get this done but I can't get myself out the door. Please will you come over?' They stay 40/45 minutes' drive from here. I just said, ‘I want to, I really want to go and work—it is my responsibility', and I could not. It doesn't matter how much I wanted to do it, I just, I couldn't.” (Debbie)*

While temporary relief occurred with the use of medication like prednisone, Trish explained it is the only time “*you don't have any pain or fatigue”* but the adverse effects complicated the management of lupus. James similarly suggested managing a concoction of “*prednisone, CellCept, a Nexus tablet and Aspavor”* needed to be balanced and “*you don't just get there overnight figuring out what works and what doesn't work.”* In his case, the prednisone side effects resulted in “*sleeping four hours and if it's really bad, then it's two hours sleep,”* and “*that broken sleep was hectic in a high-performance environment.”* James' account illustrates how organizational cultures centered on sustained high performance intensified the burden of managing complex medication regimens, offering little structural accommodation for the unpredictable rhythms of chronic illness. Even with symptomatic relief, the effects of medication resulted in unanticipated consequences, as Trish explained a colleague “*almost fell asleep behind the wheel of her car, and she fell asleep at work,”* while Karen herself experienced blackouts because of “*the cocktail of medication,”* Erica once “*slipped and fell off the stairs, because of all the cortisone”* as her “*bones are weak and muscles tired from 300 days in [the intensive care unit].”* Trish raised the concern, shared by others, of co-workers understanding the impact of medication in the management of lupus:

“*Now that I'm not on prednisone I get easily tired. People don't understand the side effects. It's a stimulant, and on the high doses, it was sometimes like 12 to 16 tablets a day*.”

Fatigue also played a role in how participants' attitudes toward their work was shaped. In most cases participants described themselves as previously being energetic and highly driven. James shared that after an extended leave of absence, “*up until that stage”* he was “*an A-type personality”*. Similarly, Karen described herself as “*a go-getter”* but as the lupus progressed, she found herself asking three types of questions regarding work: “*[whether] I want to do this? Do I have to do this? Must I do this?”* For her it was that “*fatigue equals procrastination”* and that inevitably “*procrastination becomes part of your life.”*

Even with fatigue ever present, participants wanted to show their ability was not impeded to the detriment of their health management. Karen explained that much to the dissatisfaction of her doctors, she had her “*laptop in hospital because there was a job to do.”* James said, “*I made them take me to work. I could barely walk in and barely walked out. I wanted to go to work, I needed to go to work.”* Debbie tried to explain this as “*stubbornness or denial or that you don't want to look weak in front of other people.”* For all participants there were common sources of “relief”. Erica found that when she was allowed to “*work half day,”* then “*it worked out for my good.”* Similarly, Debbie found “*working remotely on a more flexible schedule”* gave her time to “*finish work at my own pace.”* Trish was reassured when her manager showed empathy by suggesting, “*‘If you don't feel well don't come to the office; you can work from home'.”* Notably, however, these instances of relief were contingent on individual managerial discretion rather than formalized organizational policy. This structural precarity meant that access to accommodations was unevenly distributed and vulnerable to changes in line management, highlighting the absence of systemic workplace provisions for employees with invisible chronic illnesses.

Although fatigue was a prominent physical symptom, the mental health impacts of lupus were interwoven with the experiences of exhaustion. The unrelenting fatigue piled on the mental health challenges, creating a cyclical struggle where each aspect intensified the experience of the other.

### 4.2 Mental health cycles

Participants commonly experienced depression, anxiety and other mental health issues, and they described a necessary resilience to overcome these issues. Karen had many “*moments where I go into my deep dark hole and suffer from depression,”* while Erica shared not “*feeling positive”* because of lupus and her depression. Similarly, Kim acknowledged her “*depression is related to the lupus”* and contributed to her loneliness. James also recognized that “*psychological stability”* was “*a big thing”* for people with SLE. As participants withdrew from interactions with others as a coping mechanism to manage their mental health, one common but unintentional outcome was isolation. Participants considered mental health management as critical as managing physical symptoms and invariably had to find ways to do more than just cope to stay “emotionally balanced.”

“*I've got to say to [the doctor], ‘Right I'm admitted', and then spend a few days moving my meds to get me to a better place. Your mental health plays a huge role with your physical health. I cannot see how people can say they have lupus and not have a mental health issue. Lupus is a very lonely disease.” (Karen)*

The emotional burden increased with the side effects of some treatments. Participants tried to maintain a positive facade in public to avoid scrutiny or burdening others. James described it as a duality in his existence because there is “*the face I show and the face I hide.”* This performance of wellness was not merely a personal choice but a rational response to workplace environments in which visible illness risked professional marginalization, with organizational cultures that equate presence and positivity with competence, effectively compelling concealment. Similarly, Erica shared that “e*verybody in the hospital says, ‘ooh, you're so positive' but do they think I love to come through theatre 12 times a year?”* One of the most telling findings was related to the effort to maintain normalcy and conceal the extent of their struggles. Erica felt that no matter what encouragement she received from her doctors to “*get the old Erica back,”* there was a mental toll on her. In her case, she tried to keep her “*life really private except for the few friends”* she confided in. She explained that ignorance was “*the biggest challenge we face in any work situation”* as others questioned “*‘why don't doctors just give you this medication?' Or ‘Why don't you eat better and start yoga and Pilates?”'* Kim also provided insight into the despair experienced on a regular basis:

“*My psychologist said people don't realize when you get up in the morning and go to work, it does not mean that you have a good day; it means you have a day that you manage to hide how bad you're feeling. So, you only have bad days; others have good days. For a lupus person, it's always having a bad day. At times, you're strong enough to hide your bad day.”*

The uncertainty about health outcomes was distressing. Kim acknowledged that stress was her “*main cause or trigger for the lupus to flare.”* Moreover, participants seldom shared their fears and anxiety about disappointing family members and work colleagues to avoid adding to the emotional burden of their care. Erica explained, “*It's not only us with the lupus that carry a package.”* According to Erica, others could only understand the impact of lupus “*once you go through it yourself, then you see other people through other eyes.”* Battling through the uncertainty resulted in her “*losing myself in the process”* and questioning who she was.

“*In your heart, there are fears you normally don't describe to anybody. Am I going to be okay? Are they going to lose me today?”*

While fatigue impacted their mental state, the associated stress fueled a vicious cycle that triggered absences from work. In such instances, mental health support, including psychologists, mentors and a strong home support system, was crucial for managing the emotional and psychological impact of lupus.

“*I've known that stress is a key factor in flares, and in this disease, I think it's the number one thing. I've spoken to doctors about medication, anti-depressants, and they are working in collaboration.” (Debbie)*

Claiming a sense of purpose and striving to live life outside the illness was a crucial coping mechanism, and the desire for a fulfilling life was evident. According to Debbie, there was no alternative as “*it's part of life”* and “*you can't be captured in your illness where you let your illness define you”*. Similarly, Karen shared that for the last 2 years she had been “*dealing with a disease in the background”* exacerbated by the “*strain at work and home,”* which contributed to her “*emotional grief.”* Yet, as far as she was concerned, it was her “*living conditions.”* The strain of managing work and personal commitments were also discomforting and emotionally exhausting, requiring a comprehensive mental health care approach. The interplay between lupus and depression was apparent, as it created a psychological feedback loop that created a downward spiraling experience when left unchecked or untreated.

“*My lupus makes my depression worse, and my depression makes my lupus worse, and it's like it's a circle that I battle to break out of.” (Kim)*

These findings highlight the pervasive impact of fatigue and the mental health issues faced by individuals with SLE. Participants demonstrated how living with lupus poses multifaceted challenges where fatigue is inextricably linked with personal mental health struggles. Their experiences call for comprehensive support systems that address both physical and emotional health of those with invisible disabilities. Importantly, these individual experiences were not produced in isolation but were systematically shaped by rigid workplace structures, the absence of formalized accommodations policy, and organizational cultures that privilege consistent performance and penalize visible vulnerability. The findings therefore reveal that addressing the employment challenges of individuals with SLE requires structural and organizational transformation, not only individual resilience or clinical management.

## 5 Discussion

The findings of this study indicate that individuals with Systemic Lupus Erythematosus (SLE) engage in multiple paradoxes in their professional lives where they need to balance productivity and health, independence and support, and disclosure and concealment. The ever-present interplay of pervasive fatigue and mental health cycles not only disrupts daily functioning but also exposes structural barriers within workplace environments. The findings concur with the existing literature on chronic illness in the workplace, particularly the challenges faced by individuals with invisible disabilities (7, 15).

### 5.1 Paradox theory and social model of disability in context

Recalling Smith and Lewis (12) definition of paradoxes as persistent contradictions between interdependent elements that cannot simply be resolved, but must be continuously managed, the findings revealed three primary paradoxes that participants navigate daily: productivity vs. health management, independence vs. support needs, and disclosure vs. concealment (see Table 1 for themes linked to paradoxes). These tensions exemplify what Waldman et al. (38) describe as competing demands that require dynamic responses rather than static solutions.

The productivity-health paradox emerged as particularly significant, with participants describing the constant struggle to meet workplace expectations while managing unpredictable symptoms. This extends beyond theoretical constructs to reflect the lived experiences documented by Booth et al. (10) where fatigue creates fundamental conflicts with standardized productivity metrics. The pervasive nature of SLE-related fatigue described by the participants aligns with Cleanthous et al. (27) research demonstrating how fatigue impacts not only physical capacity but cognitive function and emotional regulation—dimensions that are crucial for workplace performance but often invisible to employers and colleagues.

The independence-support paradox manifested as participants frequently grappled with the internal and external pressures to demonstrate self-sufficiency while managing a condition that often requires external support. This aligns with the work of Abney et al. (7) and Kirk-Brown and Van Dijk (15), highlighting how self-reliance is often privileged in professional settings, reinforcing ableist norms that equate support-seeking with diminished competence. At the same time, the reliance on external support was not always voluntary. Several participants described situations where their symptoms forced them to seek assistance, whether from colleagues, managers or family members. This reflects a fundamental contradiction faced by individuals with SLE: while independence is highly valued, the realities of the disease often necessitate assistance. However, this need for support is frequently met with skepticism or a lack of understanding in workplace environments, further reinforcing exclusionary practices (8).

The disclosure paradox revealed in the findings mirrors the tensions identified in previous studies of invisible disabilities (9), where individuals engage in a “constant cost-benefit analysis” of revealing their condition. This paradox is exacerbated by what Calderón et al. (37) identified as the cognitive impairments associated with SLE, which create additional barriers to effective self-advocacy. The cyclical relationship between fatigue and mental health observed in our participants reflects broader findings about the bidirectional impact of physical symptoms and psychological wellbeing in chronic illness (6).

The Social Model of Disability (13, 52) provides essential context for understanding how these paradoxes are intensified by workplace structures rather than merely by the condition itself. Our findings revealed that participants encountered systemic barriers including inflexible attendance policies, standardized performance metrics and workplace environments that exacerbated symptoms. This aligns with Goering (39) conceptualization of disability as primarily a social construction rather than an individual limitation. The participants' experiences of “benevolent marginalization” (11), where employers offered options without meaningful accommodations, exemplifies what Ubel et al. (40) term the “disability paradox”—the disconnect between external perceptions and lived experiences of chronic illness.

These findings support broader calls for workplace reform, emphasizing that disability inclusion should not be an individual's burden but a shared responsibility within organizations and society at large (22).

### 5.2 Implementation framework: psychological safety and structural adaptation

The study culminates in a practical implementation framework that moves beyond theoretical principles to outline specific interventions. Drawing on Caruso Mazzolani et al. (6) findings regarding quality-of-life impacts, there are five organizational practices that can significantly improve workplace experiences for individuals with SLE:

1. Flexible performance evaluation systems that account for symptom fluctuation
2. Adaptable physical and digital work environments
3. Communication protocols that accommodate varying energy levels
4. Leadership training specifically addressing invisible disabilities
5. Structural accommodations integrated into standard operations rather than exceptional measures

This implementation framework transforms theoretical understanding into organizational change strategies, providing a distinctive contribution that bridges conceptual analysis and practical application. The implementation of flexible performance evaluations, adaptable work environments and leadership training on invisible disabilities would represent practical applications of Oliver (13) vision for implementing the social model through concrete organizational changes. By documenting both the paradoxes faced by individuals and the structural barriers they encounter, this study establishes a comprehensive approach to workplace inclusion that addresses both individual experiences and institutional practices.

### 5.3 Limitations and future research

While this study provides rich qualitative insights on invisible disabilities, several limitations warrant acknowledgment. The sample is necessarily small and draws exclusively on the perspectives of individuals with SLE, meaning that organizational viewpoints are absent from the analysis. Future research would benefit from incorporating the perspectives of managers, supervisors, and HR professionals with experience in supporting employees with invisible disabilities. Such perspectives would not only deepen understanding of the structural and policy dimensions identified in this study but would also help refine and validate the implementation framework proposed here. Furthermore, a more diverse sample as well as longitudinal studies may be useful in examining how workplace adaptations, or the lack thereof, influence the long-term employment trajectories of individuals with chronic illnesses. Aspects such as the role of cultural context and policy variance may also be considered. Further studies could explore what shifts occur in the work-life cycle of employees with SLE or investigate the disclosure paradox among those with invisible disabilities more broadly.

## 6 Conclusion

This study contributes to the literature on chronic illness and workplace inclusion by examining the interplay between fatigue, mental health and employment outcomes for individuals with SLE. The findings highlight the paradoxical tensions that individuals navigate and illustrate how structural barriers reinforce workplace exclusion. Fatigue is not just a physical limitation but a pervasive condition that affects cognition, emotional wellbeing and professional stability. This requires greater employer awareness and accommodations that must transcend standardized disability policies. Additionally, the cyclical relationship between fatigue and mental health further complicates workplace experiences, as unaddressed psychological distress exacerbates physical symptoms, making flexible work arrangements and psychological support essential. Without targeted interventions, employees with chronic illnesses remain at risk of professional instability.

Disclosure is a particularly complex and difficult decision, with many individuals fearing stigma and discrimination if they reveal their condition. This reluctance is reinforced by norms that discourage dialogue about chronic illness, isolating affected employees further. Workplaces must recognize that structural barriers and not just individual impairments are the primary drivers of workplace exclusion. The integration of Paradox Theory and the Social Model of Disability provides both explanatory power for understanding the complex experiences of employees with SLE and prescriptive guidance for creating workplaces with adaptable environments that foster inclusion and support.

Fostering inclusion in the workplace requires proactive efforts from employers. By recognizing both the internal tensions individuals navigate and the external barriers they encounter, organizations can develop comprehensive approaches to supporting employees with invisible disabilities while benefiting from their valuable contributions to the workforce. Implementing flexible work arrangements, such as modified schedules and energy management strategies, can help accommodate the fluctuating nature of chronic illnesses. Providing mental health support systems that acknowledge the psychological burden of chronic illness can further enhance employee wellbeing. Additionally, clear and equitable disclosure policies can create a safer environment for employees to seek support without fear of professional repercussions. Through recognizing the complexities of chronic illness and adopting a more equitable and proactive approach, workplaces can shift from being sites of exclusion to becoming spaces of support and empowerment. The integration of inclusive policies and empathetic leadership is essential in ensuring that individuals with SLE and other invisible disabilities are not only accommodated but valued as full contributors to the workforce.

## Data Availability

The data that support the findings of this study are not openly available because of data containing potentially identifying or sensitive participant information. Requests for further disclosure can be directed to Aden Williams aden@sun.ac.za—Ethics Committee officer. Requests to access the datasets should be directed to aden@sun.ac.za.
